# Educational Escape Rooms for Infection Prevention and Patient Safety Training in Medical and Nursing Education: A Systematic Review and Meta-Analysis

**DOI:** 10.7759/cureus.105802

**Published:** 2026-03-24

**Authors:** Anupinder Thind, Navita Aggarwal, Pravin M Pisudde

**Affiliations:** 1 Department of Physiology, All India Institute of Medical Sciences (AIIMS), Bathinda, IND; 2 Department of Anatomy, All India Institute of Medical Sciences (AIIMS), Bathinda, IND; 3 Department of Community Medicine, ESIC Medical College, Hyderabad, IND

**Keywords:** escape room, gamification, healthcare training, infection prevention, knowledge outcomes, patient safety, simulation-based education

## Abstract

Educational escape rooms have emerged as innovative tools for infection prevention and patient safety training, yet their overall effectiveness remains unclear. The existing literature shows substantial variation in instructional design, learner populations, and measured outcomes, highlighting the need for a systematic evaluation of their impact on knowledge, behavioral readiness, and process performance. To address this, we conducted a systematic review and meta-analysis in accordance with Preferred Reporting Items for Systematic Reviews and Meta-Analyses (PRISMA) guidelines. Multiple databases were searched without date restrictions, and studies were included if they employed escape room-based training for infection prevention or patient safety and reported quantitative outcomes. Risk of bias was assessed using design-specific tools, and meta-analyses were performed for outcomes reported by at least two independent studies. Twelve studies met the inclusion criteria. Meta-analysis of immediate post-training knowledge revealed a statistically significant improvement associated with escape room participation (pooled standardized mean differences (SMD) ≈ 0.86; 95% confidence interval (CI) ≈ 0.33-1.39), despite substantial heterogeneity (I² > 80%). Knowledge retention over 1-3 months showed a positive but less precise effect (pooled SMD ≈ 0.72; 95% CI ≈ −0.02-1.45). Qualitative synthesis indicated consistent benefits in engagement, self-efficacy, perceived preparedness, and teamwork, alongside favorable trends in process measures such as procedural adherence and safety behaviors; however, most of these outcomes were derived from uncontrolled studies. Overall, escape room-based interventions appear to enhance immediate knowledge in infection prevention and patient safety, with promising yet uncertain effects on knowledge retention, behavioral readiness, and process outcomes. While the findings support their educational value, methodological limitations in the existing evidence warrant cautious interpretation and further rigorous investigation.

## Introduction and background

Educational escape rooms have emerged as one of the rapidly growing instructional modalities in the field of health profession education, as educators seek innovative methods that enhance learner engagement, deepen cognitive processing, and promote performance in complex clinical scenarios for teams [1]. Rooted in experiential learning and gamification theory, these interventions combine time-limited puzzle-driven tasks with collaborative problem-solving and structured debriefing to elicit active participation and repeated retrieval of key concepts [2]. The combination of narrative framing, escalating challenges, and immediate feedback aims to enhance intrinsic motivation and situational awareness, achievements that are difficult to realize through conventional lecture formats, yet critical for training in high-risk domains [1,2]. Thus, escape room-based education has been adopted across diverse settings as a means of reinforcing such competencies that require not only knowledge but also coordinated team behavior and rapid decision-making [3].

Infection prevention and patient safety are domains in which such competencies are particularly salient. Healthcare systems continue to face persistent threats from multidrug-resistant organisms (MDROs), healthcare-associated infections, sepsis-related deterioration, and failures in the early recognition and escalation of clinical instability [3]. Despite widespread dissemination of guidelines and bundles, persistent variability in adherence to hand hygiene, isolation precautions, sepsis pathways, and incident reporting processes indicates that traditional educational strategies do not reliably achieve the behavioral reliability needed to reduce preventable harm [3,4]. Didactic sessions, e-learning modules, and unstructured clinical exposure provide limited opportunities for deliberate practice, teamwork, and reflection under realistic cognitive loads, whereas high-fidelity simulation may be resource-intensive and not routinely available [4]. Intensified interest in innovative educational formats, therefore, has focused on those that can efficiently combine knowledge acquisition with behavioral rehearsal and team coordination.

Specifically, escape room-based interventions have been proposed as one such format because they embed infection prevention and patient safety content within tightly structured scenarios that require participants to communicate, prioritize information, and apply protocols in order to "solve" the case [5]. In this configuration, tasks such as selecting appropriate personal protective equipment, interpreting sepsis criteria, or executing isolation precautions become integral steps in progressing through the game. Early reports from single institutions suggest that these activities may improve short-term knowledge, self-efficacy, and perceived readiness, and some quality improvement (QI) projects have suggested potential effects on process measures such as bundle adherence or event reporting behaviors [5,6]. However, these evaluations are dispersed across multiple disciplines, settings, and study designs, with heterogeneous outcome measures and variable methodological quality, which limits the ability to draw generalizable conclusions about effectiveness [6].

Although escape room-based learning shares certain characteristics with other simulation-based and gamified educational approaches, it represents a distinct instructional format. Unlike traditional simulation exercises, which typically focus on predefined clinical scenarios or procedural skill practice using mannequins or simulated patients, educational escape rooms involve a sequence of collaborative puzzles and problem-solving tasks that participants must complete within a time-limited environment. This structure requires learners to integrate theoretical knowledge, clinical reasoning, teamwork, and communication skills simultaneously as they progress through interconnected challenges. As a result, escape room-based learning emphasizes cognitive engagement, teamwork dynamics, and real-time decision-making rather than isolated procedural tasks.

Despite the increasing adoption of escape room-based educational activities in healthcare training programs, the available evidence on their effectiveness remains scattered across different educational settings and study designs. In particular, the impact of these interventions on measurable outcomes such as infection prevention knowledge, patient safety awareness, teamwork competencies, and learner engagement has not been systematically synthesized. Therefore, the present systematic review and meta-analysis aims to evaluate the effectiveness of escape room-based educational interventions in healthcare education, with a specific focus on infection prevention and patient safety-related outcomes.

## Review

Materials and methods

*Review* *Design*

The PECOS framework had been predefined before screening and was in line with Preferred Reporting Items for Systematic Reviews and Meta-Analyses (PRISMA) 2020 reporting guidelines [7]. Population included healthcare learners or practitioners receiving infection prevention or patient safety education. Exposure included an educational escape room or escape room-style gamified activity focused on infection prevention, sepsis, antimicrobial resistance, outbreak preparedness, or patient safety processes. Comparator included traditional teaching, alternative educational formats, or baseline values in pre-post designs. Outcomes relevant to this review include quantitative measures of knowledge, skills, attitudes, self-efficacy, teamwork, behavior, and patient safety indicators. Eligible study designs consisted of RCTs, quasi-experimental comparisons, pre-post evaluations, cross-sectional surveys reporting quantifiable outcomes, and descriptive simulation case series reporting data that could be extracted.

The review protocol, including the PECOS eligibility criteria, screening strategy, and planned analytical framework, was predefined before the screening process to ensure methodological transparency and minimize selection bias in accordance with PRISMA methodological recommendations.

*Inclusion* *and* *Exclusion* *Criteria*

Studies were included if they evaluated an escape room-based educational intervention related to infection prevention or patient safety and reported at least one quantitative outcome related to knowledge, skills, attitudes, behavior, or clinical safety metrics. Eligible participants included students or staff in any clinical or academic setting related to healthcare. Eligible study designs included randomized controlled trials (RCTs), quasi-experimental cohorts, pre-post designs, cross-sectional surveys, and descriptive simulation series. The studies were excluded if the escape room did not target infection-related or patient safety learning, if outcomes were solely qualitative, if the activity was purely recreational, or if adequate quantitative data were not reported. Opinion pieces, reviews, and non-health disciplines were also excluded.

*Database* *Searching* *Strategy*

A detailed search strategy was elaborated using Boolean operators and controlled vocabulary (MeSH/Emtree/CINAHL Headings). Six databases were systematically searched: PubMed, Embase, CINAHL, Web of Science, Scopus, and ERIC/PsycINFO. The search strategy combined three concept clusters (escape rooms/gamification, infection prevention/patient safety/sepsis, and health professions education/simulation) connected with AND. In each database, synonyms, truncation, and proximity operators were adapted. There were no date restrictions set. Reference lists and citations of the included studies were screened manually. The final database search was completed in March 2025. All retrieved records were exported into a reference management file, and duplicates were removed before screening.

Study selection followed a multistage screening process consistent with PRISMA 2020 guidance. First, titles were screened to remove clearly irrelevant publications such as non-healthcare topics, editorials, opinion pieces, and conference announcements. Second, abstracts of potentially relevant records were reviewed to determine whether the study described an escape room-based educational intervention related to infection prevention or patient safety and reported quantitative outcomes. Third, full-text articles were retrieved and evaluated in detail against the predefined inclusion and exclusion criteria. During full-text review, studies were excluded if they lacked extractable quantitative outcomes, did not involve healthcare learners or practitioners, or did not address infection prevention or patient safety content. Screening decisions were performed independently by two reviewers, and disagreements were resolved through discussion and consensus (Table 1).

**Table 1:** Search strings utilized

Database	Search string (final strategy, concept block form)
MEDLINE (PubMed)	("Escape Rooms"[Mesh] OR "escape room"[tiab] OR "escape-room"[tiab] OR "escape game*"[tiab] OR (gamif*[tiab] AND ("serious game*"[tiab] OR "game-based learning"[tiab]))) AND (("Infection Control"[Mesh] OR "Infection Prevention and Control"[tiab] OR "infection prevention"[tiab] OR "multidrug-resistant"[tiab] OR "MDRO*"[tiab] OR "sepsis"[Mesh] OR "patient safety"[Mesh] OR "medical errors"[Mesh] OR "event reporting"[tiab] OR "incident report*"[tiab])) AND (("Education, Medical"[Mesh]OR"Education, Nursing"[Mesh]OR"Students, Medical"[Mesh]OR"Students, Nursing"[Mesh]OR"in-service training"[Mesh]OR"continuing education"[tiab]OR"healthcare worker*"[tiab]OR"clinical staff"[tiab]))
Embase (Elsevier)	('escape room'/exp OR 'escape room':ti,ab OR 'escape-room':ti,ab OR 'escape game*':ti,ab OR (gamification:ti,ab OR gamified:ti,ab) AND ('serious game*':ti,ab OR 'game based learning':ti,ab)) AND (('infection control'/exp OR 'infection prevention':ti,ab OR 'infection prevention and control':ti,ab OR 'multidrug resistant organism*':ti,ab OR 'mdro*':ti,ab OR 'carbapenemase producing enterobacteriaceae'/exp OR sepsis/exp OR 'patient safety'/exp OR 'medical error'/exp OR 'incident report*':ti,ab) AND ('medical education'/exp OR 'nursing education'/exp OR 'health personnel'/exp OR 'healthcare worker*':ti,ab OR 'resident physician*':ti,ab OR 'nursing student*':ti,ab)) NOT (animal*/exp NOT human*/exp)
CINAHL (EBSCOhost)	(MH "Escape Rooms" OR TI ("escape room*" OR "escape game*") OR AB ("escape room*" OR "escape game*" OR gamification OR "game-based learning")) AND ((MH "Infection Control+" OR MH "Infection Prevention" OR TI ("infection prevention" OR "infection control" OR "multidrug resistant" OR MDRO* OR sepsis OR "patient safety" OR "adverse event reporting") OR AB ("infection prevention" OR "infection control" OR "multidrug-resistant" OR MDRO* OR sepsis OR "patient safety" OR "incident report*"))) AND ((MH "Education, Nursing, Continuing" OR MH "Education, Nursing, Graduate" OR MH "Education, Medical, Graduate" OR MH "Inservice Training" OR TI ("nursing education" OR "medical education" OR "staff training" OR "continuing education") OR AB ("clinical education" OR "professional development" OR "simulation training")))
Web of Science Core Collection	TS = (("escape room*" OR "escape-room*" OR "escape game*" OR ("serious gam*" NEAR/3 learn*) OR (gamif* NEAR/3 (educat* OR train*))) AND (("infection prevent*" OR "infection control" OR "sepsis" OR "antimicrobial stewardship" OR "multidrug resistant" OR "multidrug-resistant" OR "patient safety" OR "medical error*" OR "incident report*" OR "event report*") AND ("medical education" OR "nursing education" OR "clinical education" OR "simulation-based education" OR "healthcare worker*" OR "resident physician*" OR "nursing student*")))
Scopus	TITLE-ABS-KEY ( "escape room*" OR "escape-room*" OR "escape game*" OR ( gamif* W/3 (educat* OR train* OR learn*) ) ) AND TITLE-ABS-KEY ( "infection prevent*" OR "infection control" OR "multidrug resistant organism*" OR mdro* OR sepsis OR "patient safety" OR "adverse event" W/3 (report* OR detect*) ) AND TITLE-ABS-KEY ( "medical education" OR "nursing education" OR "health professions education" OR "simulation train*" OR "professional development" OR "in-service training" OR "continuing education" ) AND ( LIMIT-TO ( DOCTYPE, "ar" ) OR LIMIT-TO ( DOCTYPE, "cp" ) )
ERIC/PsycINFO	(AB("escape room" OR "escape-room" OR "escape game*" OR ("game-based learning" OR "serious game*") AND gamif*) AND AB("infection prevention" OR "infection control" OR "patient safety" OR "medical error*" OR "sepsis" OR "outbreak" OR "pandemic") AND AB("health professions education" OR "nursing education" OR "medical education" OR "clinical simulation" OR "interprofessional education" OR "continuing professional development")) OR (DE "Gamification" AND DE ("Patient Safety" OR "Medical Education" OR "Nursing Education") AND TI ("escape room*" OR "escape game*"))

*Data* *Extraction* *Protocol* *and* *Data* *Items*

Two independent reviewers extracted data using a standardized form. Extracted items included study setting; participant characteristics; escape room structure, including duration, tasks, and educational focus; comparator conditions; outcome measures, including instrument type, scale, and timing; quantitative results, including sample size, means, standard deviations (SDs), proportions, and confidence intervals (CIs); and implementation notes. Data needed for meta-analysis, including group sizes, means, SDs, pre-post values, or effect estimates, were collected systematically whenever available. Data on risk of bias consistent with the design of the studies were also extracted.

*Bias* *Assessment* *Protocol*

The plan for assessing risk of bias in this review was developed a priori using a design-specific framework, prior to the selection of eligible studies. Randomized controlled trials were assessed using the Cochrane Risk of Bias 2 (RoB 2) tool [8], while non-randomized comparative intervention studies (e.g., quasi-experimental designs with a comparison group) were evaluated using the Risk Of Bias In Non-randomized Studies (ROBINS-I) tool [9]. Single-group pre-post and before-after quality improvement studies were appraised using the Joanna Briggs Institute (JBI) checklist for quasi-experimental studies [10]. Cross-sectional survey designs were assessed using the AXIS tool [11].

All risk-of-bias assessments were conducted independently by two reviewers, with disagreements resolved through consensus. Each study was categorized as having low risk of bias, some concerns, or high risk of bias based on the pattern of domain-level judgments within the appropriate instrument.

Grading of Recommendations Assessment, Development and Evaluation (GRADE) Certainty Assessment

For each outcome, the certainty of the evidence was evaluated using the GRADE approach [12]. Randomized trials were initially rated as high-certainty evidence, whereas non-randomized study designs were initially rated as low-certainty evidence. Downgrading was performed for risk of bias, inconsistency, indirectness, imprecision, and publication bias, while upgrading for large effect sizes in non-randomized studies was considered where appropriate. Final certainty ratings were classified as high, moderate, low, or very low based on reviewer consensus.

*Meta*-*Analysis* *Protocol* 

Meta-analysis was planned if at least two studies reported sufficiently comparable quantitative outcomes. Continuous outcomes were synthesized as Hedges g with random-effects models. Where appropriate, dichotomous data were pooled using either a risk ratio or an odds ratio. The heterogeneity of the studies was assessed using the I² and τ². Whenever possible, sensitivity analyses excluded high-risk studies. Funnel plots were generated if ≥10 studies contributed to an outcome. All analyses were performed in Meta-Analysis Online Software [13] according to its standard operating procedures with respect to effect size entry, model selection, and output generation.

Because the included studies differed in design, participant populations, and outcome measurement scales, a random-effects model was selected a priori to account for between-study variability.

For single-group pre-post studies, effect sizes were calculated as standardized mean change (SMC), derived from the difference between post-intervention and baseline means divided by the pooled standard deviation with Hedges correction applied to adjust for small-sample bias. For randomized or comparative studies, standardized mean differences (SMD) were calculated using post-intervention group means and standard deviations.

Many primary studies did not report the correlation between baseline and post-intervention scores required for estimating the standardized mean change variance. Therefore, an assumed correlation coefficient of r = 0.50 was used, which represents a commonly applied assumption in educational meta-analyses when empirical correlations are unavailable. To assess the robustness of the results to this assumption, sensitivity analyses were conducted using alternative plausible correlations (r = 0.30 and r = 0.70), and the direction and magnitude of pooled estimates were examined across these scenarios.

Statistical heterogeneity among studies was evaluated using the I² statistic and the between-study variance parameter (τ²). Values of I² exceeding approximately 50% were interpreted as indicating moderate to substantial heterogeneity. Where heterogeneity was observed, potential sources were explored through sensitivity analyses that excluded studies with a high risk of bias and through qualitative examination of differences in study design, learner population, intervention format, and outcome measurement scales.

Results

**Figure 1:**
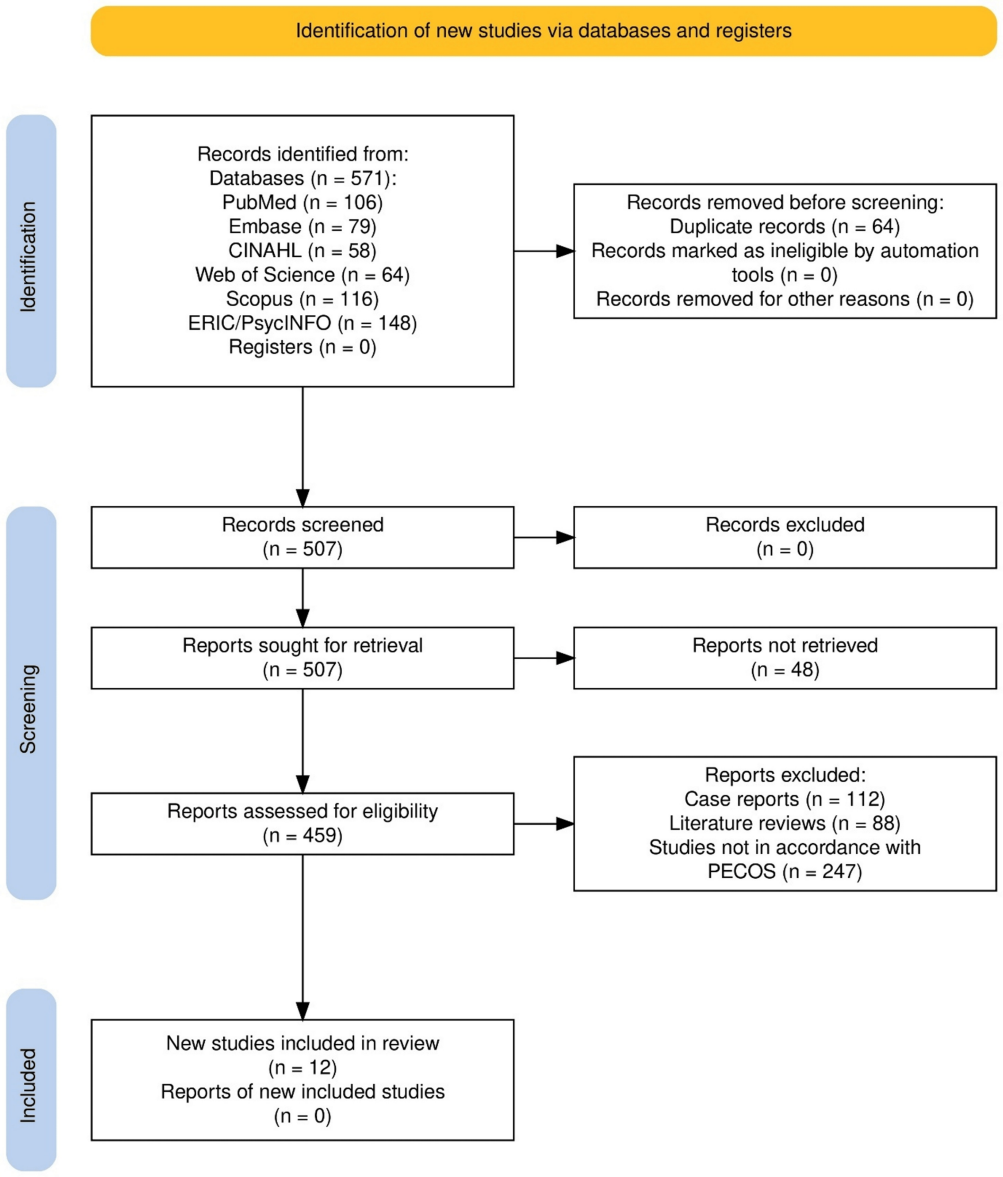
Study selection process for the review PRISMA flowchart [7] PRISMA: Preferred Reporting Items for Systematic Reviews and Meta-Analyses

Across the six electronic databases, 571 citations were retrieved, and no additional records were identified from registries (Figure 1). After eliminating 64 duplicates, 507 unique records were taken for title and abstract screening. No records were excluded at the end of this stage to retain maximum sensitivity of the initial search. All 507 records were retrieved in full text, out of which 48 could not be obtained. The remaining 459 reports went for eligibility assessment, which led to the exclusion of 112 case reports, 88 literature reviews, and 247 studies that failed to fulfill PECOS criteria [7] either due to methodological issues or on conceptual grounds. A total of 12 studies [14-25] fulfilled all the eligibility criteria and were selected.

*Bias* *Levels* *Assessed*

**Figure 2:**
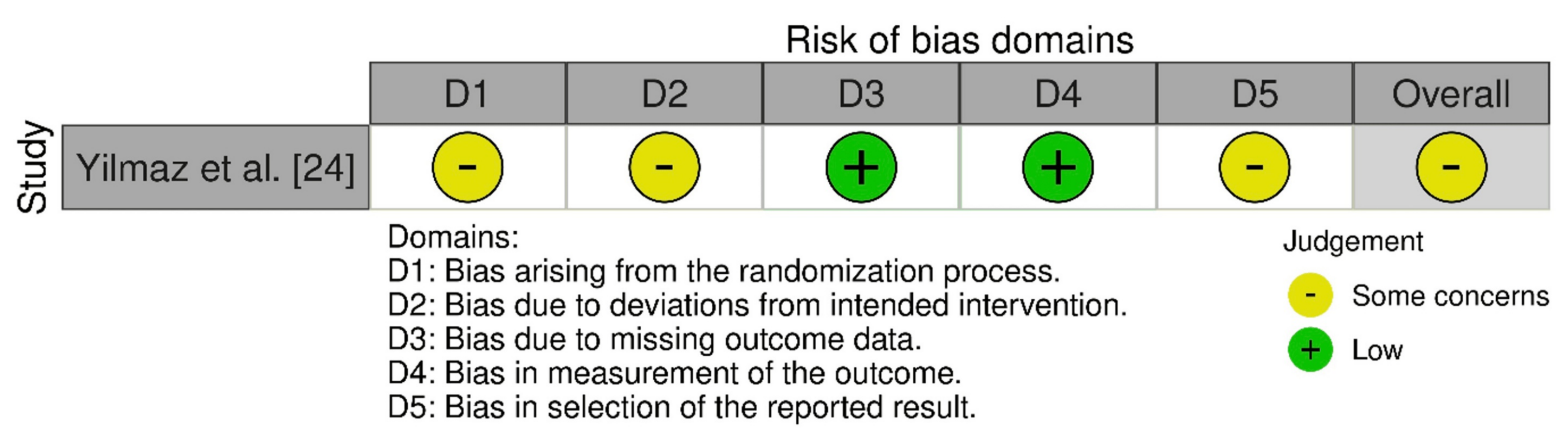
Bias assessment using the RoB 2.0 tool RoB 2 tool (licensed under the Creative Commons Attribution-NonCommercial-NoDerivatives 4.0 International License) [8] RoB 2: Risk of Bias 2

**Figure 3:**
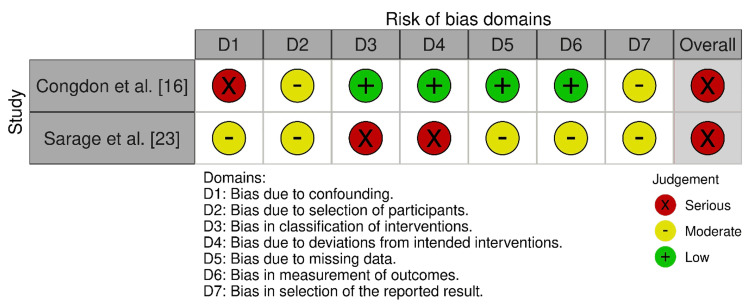
Bias assessment using the ROBINS-I tool ROBINS-I tool (licensed under the Creative Commons Attribution-NonCommercial-NoDerivatives 4.0 International License) [9] ROBINS-I: Risk Of Bias In Non-randomized Studies

Only one of the included studies was an RCT: Yilmaz et al. [24]. It presented overall concerns mainly due to unclear or inadequate randomization and risk for selective reporting (Figure 2). ROBINS-I [9] assessment indicated a generally elevated risk of bias (Figure 3). Congdon et al. [16] displayed a serious overall risk because of serious confounding and moderate selection bias, despite low risks in the intervention classification, deviations, missing data, and outcome measurements. Sarage et al. [23], on the other hand, demonstrated a serious overall risk since there were serious questions regarding intervention classification and deviations with moderate concerns across other domains.

**Figure 4:**
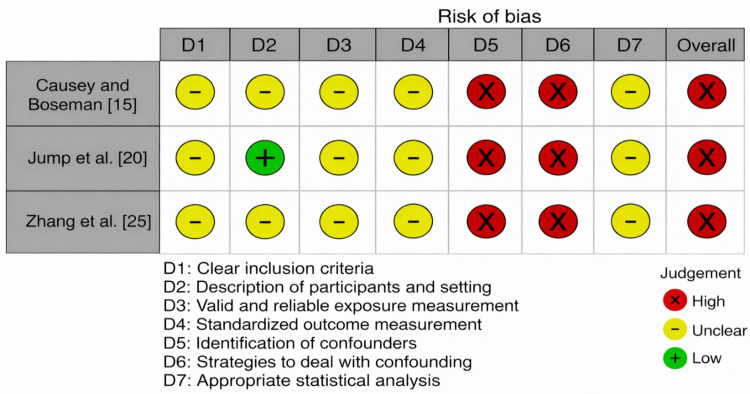
Bias assessment using the AXIS tool AXIS tool (©Latitudes Network, 2026) [11]

**Figure 5:**
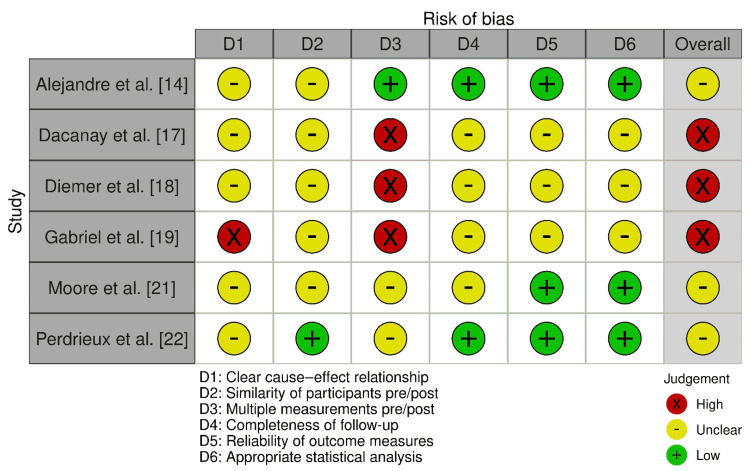
Bias assessment using JBI quasi-experimental/NIH before-after single-group pre-post and quality improvement JBI checklist for quasi-experimental studies (©Joanna Briggs Institute, 2017) [10] JBI: Joanna Briggs Institute

Causey and Boseman [15], Jump et al. [20], and Zhang et al. [25] each demonstrated a high overall risk of bias under the AXIS instrument (Figure 4) [11]. While generally acceptable exposure and outcome measurement, recurrent deficiencies in identifying and addressing confounders, taken in concert with reliance on self-report measures, contributed to uniformly elevated domain-level concerns. The quasi-experimental pre-post designs exhibited mixed risk patterns (Figure 5) [10]. Alejandre et al. [14], Moore et al. [21], and Perdrieux et al. [22] showed some concerns overall, reflecting acceptable reliability of outcome measures and analytic approaches but limited control for temporal biases and participant comparability. On the other hand, Dacanay et al. [17], Diemer et al. [18], and Gabriel et al. [19] had high overall risk related to incomplete follow-up, absence of repeated measurements, weaker analytic frameworks, and concerns about confounding and measurement reliability.

*Timeframe* *and* *Geographic* *Setting* *Assessed*

The included studies were relatively recent, spanning 2019-2025 [14-25], with the majority of those having been conducted from 2021 onward [15-17,19-24], suggesting that evidence is predominantly contemporary (Table 2). The studies were primarily from the United States [15-18,20,21,23,25], with other studies from Spain [14], France [22], Australia [21], and Turkey [24], contexts that are mostly classified as high-income or upper-middle-income.

**Table 2:** Demographic characteristics NR: not reported, QI: quality improvement, N: number, RCT: randomized controlled trial, PALTC: post-acute and long-term care, ER: escape room, IP: infection prevention, CAUTI: catheter-associated urinary tract infection, FY: fiscal year, USA: United States of America

Author ID	Year	Location	Study design	Sample size	Mean age (years)	Male/female	Follow-up
Alejandre et al. [14]	2022	Spain	Single-group pre-post + 3 months	48 pediatric residents	26.2 ± 2.03	10/38	Immediate + 3 months
Causey and Boseman [15]	2021	USA	Cross-sectional QI (conference/IP program)	189 participants (approximately, from report)	NR	NR	None (single event)
Congdon et al. [16]	2024	USA	Quasi-experimental (ER versus non-ER cohorts)	FY2020: 20 ER versus 30 non-ER; FY2021: 44	NR	NR	Behavior tracked ≈2 years
Dacanay et al. [17]	2021	USA	Before-after QI (sepsis + CAUTI)	Hospital inpatient nurses (~95% staff; N NR)	NR	NR	6 months (CAUTI); 5 versus 3 months (sepsis)
Diemer et al. [18]	2019	USA	Single-group pre-post	130 enrolled; 102 analyzed	NR	NR	Immediate post only
Gabriel et al. [19]	2021	USA	Educational QI (sepsis ER + bundle audit)	N of nurses NR (16 nurse groups)	NR	NR	≈2 months (bundle adherence)
Jump et al. [20]	2025	USA	Descriptive QI (>300 PALTC facilities)	Facility number > 300; individual N NR	NR	NR	18-month program (no fixed tests)
Moore et al. [21]	2021	Australia	Mixed-methods single-group pre-post	50 students	NR	≈11/39 (78% female)	Immediate post only
Perdrieux et al. [22]	2025	France	Single-group pre-post + 3-months	141 (44 at 3 months)	NR	NR	Immediate + 3 months
Sarage et al. [23]	2021	USA	Descriptive simulation	NR (8-10 per session)	NR	NR	None (single session)
Yilmaz et al. [24]	2025	Turkey	RCT (escape room versus lecture)	60 (30/30)	18.72 ± 0.90	11/49	1 week post-test
Zhang et al. [25]	2019	USA	Post-activity descriptive	130 enrolled; 102 analyzed	NR	NR	None

*Study* *Design*

The most common designs were single-group pre-post and descriptive/quality improvement (QI) formats [14,15,17-19,21-23,25], with only one quasi-experimental cohort comparison [16] and one RCT [24], indicating that few controlled comparative data exist.

*Sample* *Size*

Sample sizes span from moderate classroom or residency cohorts of approximately 48-60 participants [14,21,24] to larger staff education events of approximately 189 participants [15], multi-facility rollouts with less clear individual counts [17,20], and incomplete specification in some reports of learner numbers [19,23].

*Age* *and* *Gender* *Profiles*

Mean age was indicated only for residents (about 26 years [14]) and first-year nursing students (about 19 years [24]), and sex distributions were explicitly quantified in these two cohorts (female-majority samples [14,24]), with other studies omitting basic demographic breakdowns [15-23,25].

*Follow*-*Up* *Duration*

The majority of the measurements were taken as immediate post-intervention [15,18,21,23,25], while some included short-term one-week [24] or three-month [14,22] follow-up, while a few QI and behavioral studies extended the observation window to several months for clinical outcomes [17] or up to two years for reporting behavior [16] (Table 2).

*Educational* *Topic* *Focus*

Throughout the reviewed studies (Table 3), the orientation of educational content has been toward infection prevention domains such as pediatric sepsis management, multidrug-resistant organisms, device-associated infection [14,17,19-20,22], and broader constructs of patient safety, including hazard identification, event reporting, medication safety, and teamwork [15,16,18,21,23-25].

**Table 3:** Technical and statistical characteristics IP: infection prevention, PS: patient safety, ER: escape room, CI: confidence interval, ED: emergency department, MCQ: multiple-choice question, MD: mean difference, PPE: personal protective equipment, PGY-1: post-graduate year 1, MER: medical escape room, RR: risk ratio, CAUTI: catheter-associated urinary tract infection, ICU: intensive care unit, SEP-1: Severe Sepsis and Septic Shock Early Management Bundle, QI: qualitative index, SMD: standardized mean difference, MDRO: multidrug-resistant organism, PALTC: post-acute and long-term care, EVS: environmental services, AHRQ: Agency for Healthcare Research and Quality, MRSA: methicillin-resistant Staphylococcus aureus, IPE: interprofessional education, IPP: interprofessional practice, XDR: extensively drug-resistant, UV: ultraviolet, QSEN: Quality and Safety Education for Nurses, PSKT: patient safety knowledge test, KR-20: Kuder-Richardson Formula 20, RCT: randomized controlled trial, CIQ: critical incident questionnaire, SD: standard deviation

Author ID	Educational topic focus (IP/PS domain)	Learner cadre/level	Simulated clinical environment	ER design characteristics (number of puzzles/rooms; time limit)	Primary outcome domain (for review)	Outcome instrument and scoring range (test name; items; 0-X scale)	Assessment timepoint used in meta-analysis	Comparator type	Statistical analysis approach	Effect size metric for primary outcome	Effect size estimate with 95% CI	Overall conclusion assessed
Alejandre et al. [14]	Pediatric sepsis recognition and initial management (infection-related patient safety)	Pediatric medical and nursing residents	High-fidelity pediatric ED resuscitation bay	Single sepsis ER case; teams of 4-5; 60-minute session	Knowledge of pediatric sepsis management	9-item sepsis MCQ; score 0-9 (guideline-based)	Immediate post-test versus pre-test (3-month follow-up for retention)	Single-group pre-post	Wilcoxon signed-rank tests (pre versus post; pre versus 3 months)	Within-group MD; standardized mean change possible with assumed r	Pre→post MD ≈ +0.90/9; pre→3-month MD ≈ +0.45/9 (both p < 0.01; 95% CI for change not reported)	ER produced a significant, durable increase in sepsis knowledge, with partial retention at 3 months.
Causey and Boseman [15]	Infection prevention and pandemic preparedness (PPE choice, contamination spread, hand hygiene)	Mixed hospital staff (clinical and non-clinical)	Brief pandemic unit-style simulation space	10-minute ER scenario; small interprofessional groups; multiple embedded tasks/stations	Engagement/participation in infection prevention training	Attendance counts and brief Likert-type feedback; no formal test score	Immediate post-participation only (single event)	None (no pre-post, no parallel control)	Descriptive statistics only (counts, proportions, narrative feedback)	- (no analyzable primary effect metric)	No formal effect size (behavioral "rate ratio" versus usual voluntary training not estimable from available data)	ER yielded a marked increase in voluntary participation in IP education but offers no quantifiable knowledge or behavior effect.
Congdon et al. [16]	Patient safety: hazard recognition and real safety event reporting behavior	Pediatric interns (PGY-1)	Simulated pediatric inpatient/clinic setting	Single MER (patient safety ER); ~30-40 minutes; interns identify hazards and file an actual report	Behavior: filing ≥1 safety event report during training	Binary indicator (≥1 versus 0 real reports filed over follow-up) plus self-rated confidence (Likert)	2-year behavioral follow-up (FY2020 ER versus non-ER cohort)	Parallel cohort comparison (ER versus non-ER) plus within-group pre-post for attitudes	Fisher’s exact tests for behavior; independent and paired t-tests for confidence scores	RR for ≥1 event report	From 14/20 (ER) versus 13/30 (non-ER): RR ≈ 1.62, 95% CI ≈ 0.98-2.66 (borderline but meaningful)	Participation in the ER substantially increased the likelihood of filing at least one safety event report, with a moderate-to-large behavioral effect.
Dacanay et al. [17]	Infection prevention and patient safety: CAUTI prevention and sepsis SEP-1 bundle performance	Adult inpatient nurses (step-down, ICU, general wards)	Hospital units with skills stations and a team-based ER scenario	Multistation ER integrated into skills blitz; typical completion ≈42 minutes; number of puzzles not fully specified	Clinical/process outcomes: CAUTI incidence and time-critical sepsis bundle elements	CAUTI events over 6 months pre versus 6 months post; SEP-1 process measures (e.g., timely lactate draw) over 5 months pre versus 3 months post	Pre- versus post-implementation windows for CAUTI and sepsis outcomes	Uncontrolled before-after	Descriptive run charts and rate comparisons; formal tests not reported	- (no consistent effect size derivable)	Numerical effect sizes (RR/IRR) not estimable due to missing denominators and variance	ER QI bundle was associated with lower CAUTI rates and improved sepsis process measures, but the absence of analyzable statistics limits its use to narrative clinical evidence.
Diemer et al. [18]	General patient safety: hazard identification and electronic event reporting	New interns across multiple specialties	Simulated inpatient rooms	Two ER cases per team; ≈20 minutes each plus debrief	Attitudes/self-efficacy for hazard ID and reporting; hazard detection behavior during scenario	Custom confidence/comfort items (Likert-type) and count of hazards identified/reports completed	Immediate post-session versus pre-session	Single-group pre-post	Descriptive summaries; limited pre-post comparisons (no full reporting of means/SDs)	- (cannot calculate MD/SMD robustly)	No reliable CI; only directionally improved self-efficacy and hazard identification performance	ER improved self-reported comfort and practice with reporting, but incomplete numeric data preclude inclusion in quantitative synthesis.
Gabriel et al. [19]	Sepsis patient safety: adherence to evidence-based sepsis bundles	Acute care nurses	Simulated patient room with manikin	Sepsis-themed ER with multiple puzzles; ≈20 minutes per group; followed by debrief	Clinical process: sepsis bundle adherence; staff evaluation of activity	Unit-level SEP-1 bundle adherence (%) and session evaluation on a 1-5 scale	Bundle adherence compared pre- versus post-implementation (≈2-month window); evaluation immediate post-session	Uncontrolled before-after for adherence; single-group post-only for evaluation	Descriptive statistics (mean evaluation ~4.9/5); adherence trend summaries	- (no precise MD or RR extractable from published data)	Effect magnitude not quantifiable; no CI	Sepsis ER achieved very high staff ratings and was associated with improved bundle adherence, but missing numerics limit meta-analytic use.
Jump et al. [20]	Infection prevention: MDRO and enhanced barrier precautions in PALTC	PALTC frontline staff (nurses, NAs, EVS, etc.)	Table-top ER game representing a nursing home resident and environment	Printable game with 4 “rooms” × 2 puzzles (8 puzzles in total); no fixed time limit; run in education sessions	Engagement/practice of IP concepts (no formal knowledge/behavior endpoint)	None: no scored test; used as a teaching tool embedded in AHRQ MRSA program	Not applicable (no specific test timepoint)	None (no formal comparator)	Descriptive feedback and implementation reports only	-	No effect estimate or CI possible	ER-style game appears highly scalable and low-cost, reinforcing IP concepts in PALTC, but generates no extractable outcome data.
Moore et al. [21]	Interprofessional teamwork and collaboration (not IP-specific but safety-relevant)	Mixed health professions students (multiple disciplines)	Teaching/classroom setting	Single interprofessional ER followed by debrief	Interprofessional learning (knowledge/attitudes)	IPE/IPP questionnaires (multiple subscales; Likert-type)	Immediate post-test versus pre-test	Single-group pre-post	Paired statistical tests plus qualitative analysis	Could be SMD/MD for IPE scales, but not IP/PS focused	Effect sizes for IPP outcomes not reported in IP/PS terms	ER enhanced interprofessional learning outcomes; relevant context for teamwork, but not directly quantifiable for infection prevention/patient-safety endpoints.
Perdrieux et al. [22]	Infection prevention: MDRO/XDR isolation and screening	Nurses and nurse assistants in a tertiary hospital	Simulated isolation room with XDR scenario	60-minute escape session; 6 riddles (4 MDRO knowledge, 2 file/UV tasks)	Knowledge about MDRO/XDR procedures	11-item MCQ; reported as % correct	Immediate post-test versus pre-test (3-month follow-up for retention)	Single-group pre-post	Wilcoxon signed-rank; multivariable regression	MD in % correct	Pre→post MD ≈ +21.6 percentage points (approximate 95% CI +17.7 to +25.5); substantial effect; 3-month scores remained significantly above baseline	ER training produced a large, statistically robust knowledge gain in MDRO infection prevention with good 3-month retention.
Sarage et al. [23]	Medication safety and teamwork (patient safety competencies)	Junior and senior undergraduate nursing students	High-fidelity ward/patient room	Single ER simulation per group; multiple medication and teamwork puzzles; ~1 laboratory period	Medication-safety behaviors and teamwork skills	Observed performance (puzzle completion, med steps); debrief discussion; no numerical scale reported	Immediate performance during session	None (single-arm educational innovation)	Descriptive only; no inferential tests	-	No effect size or CI	ER was feasible and aligned with QSEN safety competencies, but the absence of standardized outcome measures prevents quantitative assessment.
Yilmaz et al. [24]	General patient safety (falls, ID checks, consent, med safety, etc.)	First-year baccalaureate nursing students	Skills laboratory/classroom PS scenarios	3 ER sessions; 2 games per session over 2 weeks; ER versus lecture control	Knowledge of patient safety (primary); cooperation (secondary)	PSKT: 24 MCQs, 0-24; KR-20=0.86; cooperation scale: 11 items, 11-55; α = 0.842.	Post-test (1 week post-intervention) PSKT between groups	Parallel-group RCT (ER versus traditional teaching)	Independent-samples t-tests (between groups); paired t-tests (within); Pearson correlation	SMD (Hedges g) for PSKT post-test	Using post means/SDs (17.03 ± 3.03 (ER) versus 14.53±3.56 (control), n = 30/30): g ≈ 0.75, 95% CI ≈ 0.23-1.26, favoring ER	ER produced a moderate-large improvement in patient safety knowledge versus lecture and a significant, smaller gain in cooperation scores, making it a key controlled study for meta-analysis.
Zhang et al. [25]	Patient safety engagement and hazard perception (orientation)	Intern physicians (same center as Diemer et al.)	Simulated patient room	Single QR code-driven ER embedded in intern orientation	Engagement/perceived learning about patient safety and hazards	CIQ: open-ended prompts; no quantitative scale	Post-activity only	None (post-only single group)	Qualitative thematic analysis; frequency counts of themes	-	No effect metric or CI possible	ER enhanced perceived engagement, teamwork, and hazard awareness, but only qualitative data are reported; not suitable for quantitative pooling.

*Learner* *Cadres* *and* *Simulated* *Environments*

The interventions involved both postgraduate residents and frontline clinical staff in both acute care and long-term care settings [14,16,17,19,20,22], as well as undergraduate nursing and mixed health professions students at the pre-licensure level [21,23,24]. Simulated environments predominantly recreated high-risk clinical micro-environments such as emergency or critical care bays and isolation rooms [14,16,17,19,22], along with skills laboratory or classroom implementations and table-top activities for broader staff engagement [15,20,21,23-25].

Escape Room Design Characteristics

The typical format for escape rooms used one clinical scenario per room in a structured puzzle format with time-limited team tasks generally in lengths from 20 to 60 minutes per session [14,16,19,21,22]. Some interventions used multiroom or multipuzzle formats to encode sequential clinical decisions (e.g., four rooms with eight puzzles) [17,20]. Other interventions embedded a shorter single-room format into larger orientation or skills-blitz curricula [15,18,23-25].

*Primary* *Outcome* *Domains*

Primary outcome domains were heterogeneous and included knowledge acquisition related to sepsis and multidrug-resistant organism protocols [14,22,24], real-world or process-based behavioral outcomes such as safety event reporting and bundle adherence [16,17,19], and proximal educational endpoints that include teamwork, cooperation, and engagement without direct assessment of clinical knowledge or behavior [15,18,20,21,23,25].

*Outcome* *Instruments* *and* *Scoring*

Knowledge outcomes were measured using structured multiple-choice tests with bounded score ranges (e.g., 0-9 or 0-24 scales), which allowed calculation of mean differences in absolute or percentage-correct scores [14,22,24]. Behavioral and clinical outcomes depended on binary or rate-based measures, including "at least one event report filed," catheter-associated infection rates, and compliance with sepsis bundles [16,17,19]. A number of the implementations utilized non-standardized observational performance ratings, debrief assessments, or open-ended qualitative measures, which by design ruled out derivation of continuous effect sizes [15,18,20,21,23,25].

*Assessment* *Timepoints* *and* *Comparators*

Most educational effects were assessed immediately post-intervention, although some studies included follow-up measurements at about three months to measure knowledge retention [14,22]. Comparator designs varied from single-group pre-post designs lacking external controls [14,18,19,21-23] and uncontrolled before-after QI designs for clinical indicators [17] to between-cohort comparisons for real-world behavior [16] and a single randomized parallel-group design comparing escape room training with traditional teaching [24].

*Methods* *of* *Statistics* *and* *Effect*-*Size* *Patterns*

Analytical methods mainly used non-parametric paired tests for within-group change in studies with modest sample sizes or non-normally distributed scores [14,22], while in the randomized trial, parametric t-tests and correlation analyses were used for between-group and within-group tests [24]. Behavioral outcomes were subjected to exact tests for proportions [16], and quality improvement-oriented reports remained mostly descriptive in nature [15,17,19,20,23,25]. Where quantitative effect sizes could be derived, knowledge gains were large in absolute terms, with immediate post-training mean differences of around 0.9 points on a 0-9 sepsis scale [14] and about 21.6 percentage points on an 11-item MDRO knowledge test [22], and a moderate-to-large standardized mean difference of approximately 0.75 in favor of escape room instruction in patient safety knowledge in the randomized comparison [24]. The behavioral effect of event reporting was large in magnitude, with a risk ratio estimate of about 1.6 for filing at least one report subsequent to participation, although confidence intervals narrowly crossed 1.0 [16].

*Meta*-*Analysis* *Observations*

**Figure 6:**
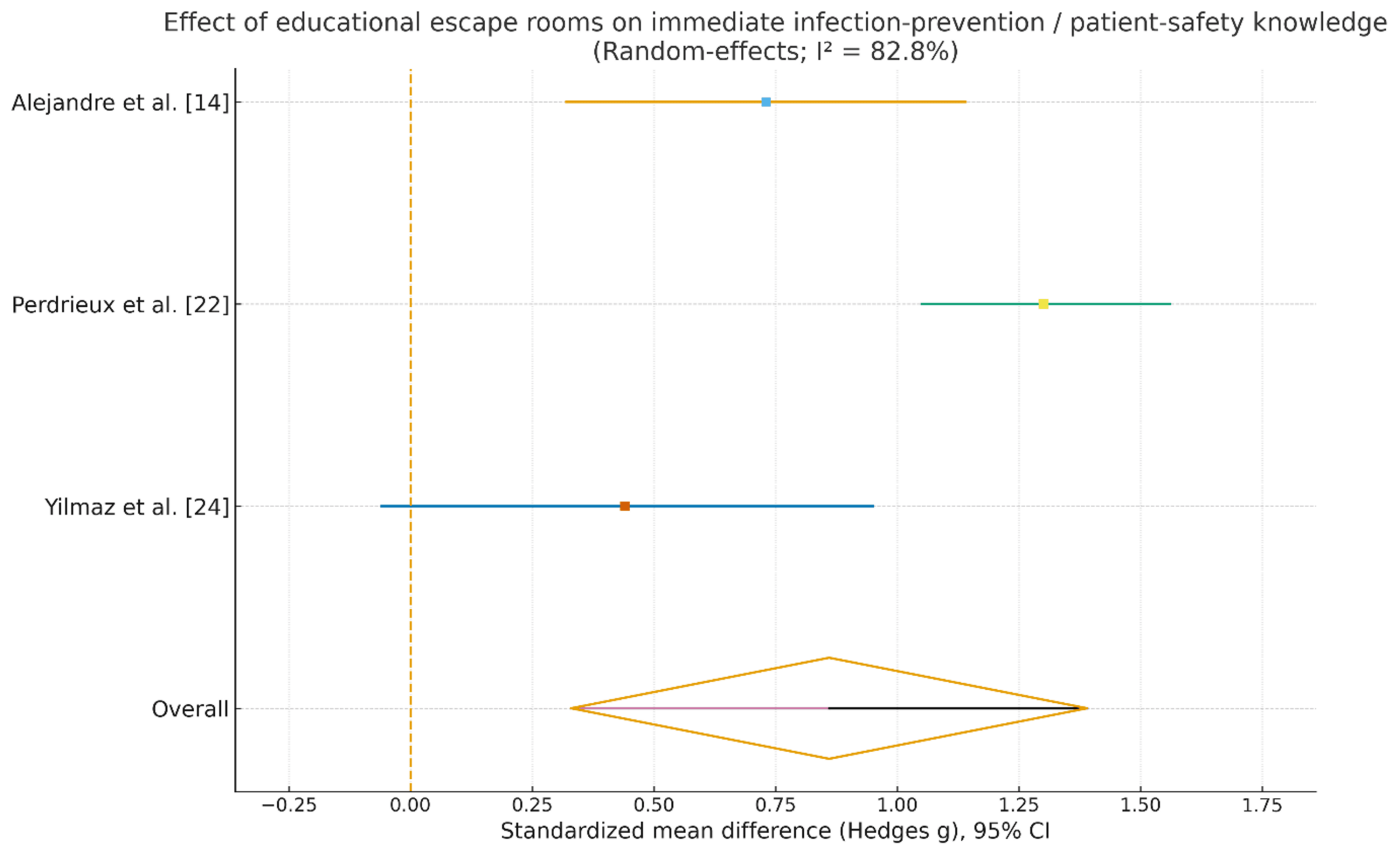
Effect of educational escape rooms on immediate infection prevention/patient safety knowledge CI: confidence interval

The forest plot in Figure 6 shows that educational escape rooms were associated with a clear improvement in immediate infection prevention and patient safety knowledge. In all three studies [14,22,24], the escape room intervention outperformed baseline, with standardized mean differences ranging from a small-to-moderate effect (g ≈ 0.44, confidence interval marginally crossing zero [24]) to a large effect (g ≈ 1.30, confidence interval completely above zero [22]). In Alejandre et al. [14], a moderate effect was found (g ≈ 0.73) with a clearly positive confidence interval. Aggregating these studies via a random-effects model led to a pooled effect size in the moderate-to-large range (g ≈ 0.86); the width of the 95% confidence interval excluded zero, suggesting that, on aggregate, escape room-based instruction significantly increased immediate knowledge scores. However, substantial statistical heterogeneity was found (I² ≈ 83%), implying large variation in the magnitude of benefit across the studies, likely due to variations in learner populations, outcome scales, and implementation of the escape rooms.

**Figure 7:**
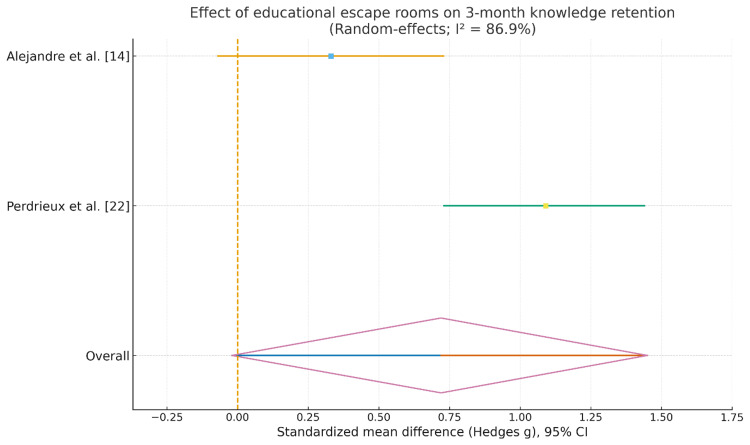
Effect of educational escape rooms on three-month knowledge retention CI: confidence interval

The second forest plot (Figure 7) addressed knowledge retention at approximately three months. Again, both included studies favored the escape room condition relative to baseline, although with disparate magnitudes. Alejandre et al. [14] reported a small positive effect at follow-up (g ≈ 0.33) with a confidence interval crossing zero, indicating statistical uncertainty, whereas Perdrieux et al. [22] demonstrated a large and unambiguously significant effect on retained knowledge (g ≈ 1.09, confidence interval entirely above zero). The pooled random-effects estimate fell within the moderate range (g ≈ 0.72), but its 95% confidence interval narrowly included zero, so the overall effect on long-term retention did not reach conventional statistical significance. Heterogeneity remained high (I² ≈ 87%), implying substantial between-study variation in long-term impact.

*GRADE* *Assessment* *Observations*

The GRADE assessment (Table 4 and Table 5) [12] showed that the highest quality evidence related to immediate post-training knowledge (three studies, including one randomized trial) indicated a moderate to large increase in knowledge scores following escape room-based infection prevention or patient safety training [14,22,24]. However, the certainty for this outcome was rated as only moderate after downgrading for risk of bias concerns and substantial statistical heterogeneity. For longer-term knowledge retention at approximately one to three months, only two non-randomized pre-post studies contributed data [14,22], and the pooled estimate had a wide CI crossing no effect, which, along with important methodological limitations and heterogeneity, resulted in a very low certainty rating. Similarly, the evidence for attitudes, self-efficacy, and perceived readiness came from uncontrolled pre-post and QI designs using self-report measures [15,17-19,21] and was rated as very low certainty despite a consistent direction of favorable findings. Behavioral and process outcomes [19-22], including bundle adherence and sepsis-related transfers, were informed solely by uncontrolled QI data with high risk of confounding and indirectness, yielding an overall certainty rating of very low.

**Table 4:** GRADE evidence profile (by outcome domain) GRADE (available under the Creative Commons Attribution-NonCommercial-ShareAlike 3.0 IGO license [CC BY-NC-SA 3.0 IGO]) [12] GRADE: Grading of Recommendations Assessment, Development, and Evaluation, CI: confidence interval, I²: I-squared statistic, No.: number, QI: qualitative index, RCT: randomized controlled trial, SMD: standardized mean difference, IPC: infection prevention and control

Outcome domain	Number of studies	Design of included studies	Study limitations (risk of bias)	Between-study variability	Indirectness of evidence	Statistical imprecision	Suspected reporting bias	Overall certainty of evidence
Immediate post-training knowledge (escape room-based infection prevention/patient safety)	3 (Alejandre et al. [14], Perdrieux et al. [22], Yilmaz et al. [24])	1 RCT, 2 single-group pre-post studies	Downgraded: some concerns in randomization and selective reporting in the RCT [24], and some concerns about the high risk of bias in pre-post designs [14,22]	Downgraded: substantial heterogeneity in effect sizes and implementation (I² > 80%)	Not downgraded: populations, interventions, and outcomes aligned with review question	Not downgraded: pooled effect estimate showed a clearly positive SMD with CI not crossing 0	Not downgraded: no clear evidence of small-study or selective publication patterns	Moderate (started at high for RCT, downgraded for risk of bias and inconsistency)
Short- to medium-term knowledge retention (≈1-3 months)	2 (Alejandre et al. [14], Perdrieux et al. [22])	Single-group pre-post with follow-up	Downgraded: some concerns in both studies, with a non-randomized design and potential temporal confounding [14,22]	Downgraded: high heterogeneity in the magnitude of retention effects	Downgraded: some differences in timing and content intensity across studies	Downgraded: confidence interval for pooled SMD included no effect	Not downgraded: no strong evidence of selective reporting beyond design limitations	Very low (non-randomized designs starting at low, further downgraded for inconsistency and imprecision)
Attitudes, self-efficacy, and perceived readiness for infection prevention/patient safety tasks	5 (Causey and Boseman [15], Dacanay et al. [17], Diemer et al. [18], Gabriel et al. [19], Moore et al. [21])	Quasi-experimental pre-post and descriptive QI	Downgraded: high or some concerns risk of bias in most studies (no control groups, incomplete follow-up, self-report measures) [15,17-19,21]	Downgraded: variability in outcome constructs, scales, and effect magnitudes	Downgraded: some interventions combined escape rooms with broader initiatives; outcomes often general rather than IPC-specific	Downgraded: small samples in several studies and absence of precise effect estimates	Downgraded: selective reporting likely, with emphasis on positive results in QI contexts	Very low (non-randomized, uncontrolled, and methodologically heterogeneous evidence)
Behavioral and process outcomes (e.g., bundle adherence, event reporting, sepsis-related transfers)	4 (Gabriel et al. [19], Jump et al. [20], Moore et al. [21], Perdrieux et al. [22])	Before-after QI and descriptive observational studies	Downgraded: serious risk of bias due to confounding, co-interventions, and uncontrolled secular trends [19-22]	Downgraded: heterogeneous process indicators and measurement strategies	Downgraded: indirect links between the escape room and downstream process or clinical metrics	Downgraded: few events and a lack of precise effect measures in several reports	Downgraded: high risk of selective reporting of favorable QI signals	Very low (highly indirect, uncontrolled QI evidence with multiple limitations)

**Table 5:** Summary of findings GRADE: Grading of Recommendations Assessment, Development, and Evaluation, CI: confidence interval, OR: odds ratio, RR: risk ratio, SD: standard deviation, SMD: standardized mean difference, ICU: intensive care unit, QI: qualitative index

Key outcome	Expected absolute effects (escape room versus comparator or baseline)	Relative effect (standardized or relative measure)	Participants (studies)	Overall certainty of the evidence (GRADE)
Immediate knowledge gain after escape room-based infection prevention/patient safety training	Across three studies, typical control or pre-intervention groups had moderate baseline knowledge scores; escape room exposure was associated with an increase of roughly 0.5-1.5 SD units in post-test scores, corresponding to a clear shift toward higher test performance [14,22,24]	Pooled standardized mean difference ≈ 0.86 (95% CI ≈ 0.33-1.39) in favor of escape room training [14,22,24]	≈ 219 participants (3 studies)	Moderate certainty
Knowledge retention at ≈1-3 months after escape room training	In two pre-post studies, follow-up scores remained higher than baseline, but the magnitude of retained gain varied, and one study showed only a small additional increase over pre-intervention values [14,22]	Pooled standardized mean difference ≈ 0.72 (95% CI ≈ -0.02-1.45), with high heterogeneity and confidence interval including no effect [14,22]	≈ 92 participants (2 studies)	Very low certainty
Attitudes, self-efficacy, and perceived readiness for infection prevention/patient safety tasks	Multiple studies reported improvements in self-rated confidence, perceived preparedness, and attitudes toward infection prevention and sepsis care after escape room participation, but effects were based on uncontrolled pre-post comparisons and self-report scales [15,17-19,21]	Direction of effect generally favored escape room interventions; no robust pooled estimate was available because of heterogeneous measures and incomplete statistics [15,17-19,21]	>300 participants across 5 studies	Very low certainty
Behavioral and process outcomes (bundle adherence, event reporting, sepsis-related ICU transfers, related safety processes)	Individual QI reports described post-intervention improvements in process indicators (e.g., increased adherence to sepsis bundles, increased reporting of safety events, reductions in sepsis-related ICU transfers), but these changes occurred in uncontrolled before-after contexts with possible co-interventions [19-22]	Effects consistently favored the post-intervention periods qualitatively, but relative measures (RR/OR) could not be reliably estimated across studies due to missing denominators and concurrent system changes [19-22]	Several hundred participants and multiple units across 4 studies	Very low certainty

Discussion

The integration of escape room pedagogy into health professions education represents a larger movement toward active learning and experiential modalities capable of targeting cognitive, behavioral, and contextual determinants of clinical performance. As healthcare environments grow increasingly complex, educators have pursued approaches that approximate the cognitive load, ambiguity, and interdependent workflows representative of authentic clinical environments. Previous research in high-acuity disciplines has established that variation in frontline assessment and performance often results from limitations in situational awareness, communication, and the reliability of procedural performance rather than knowledge deficits in isolation [26-29]. In this context, educational escape rooms have attracted interest due to their potential to immerse learners in interactive and immersive scenarios requiring rapid interpretation, synthesis of information, and coordination as teams, attributes integral to infection prevention and patient safety competency.

Preliminary studies on escape room-based learning suggested that such environments provide a unique blend of gamification, narrative immersion, and collaborative puzzle solving that improves learner engagement over more traditional didactic approaches [27]. Meta-analytic evidence similarly suggests that learning via escape room formats may confer cognitive, motivational, and behavioral advantages over more conventional modalities, particularly under conditions in which learners must integrate procedural steps with conceptual understanding in a time-constrained manner [28]. Applications within advanced nursing and medical curricula further showed that the structured challenge-reward cycle intrinsic to escape rooms serves to reinforce adult learning principles, facilitating deeper processing and retention of complex subject matter [29].

Related research in game-based and gamified learning environments has shown that carefully designed tasks can stimulate emotional activation, promote attentional focus, and reduce performance anxiety, particularly in simulation-based laboratories [30,31]. These features are pertinent to infection prevention and patient safety competencies, wherein learners must integrate technical steps with vigilance, communication, and timely decision-making. Scoping reviews have highlighted that escape rooms can also function as flexible platforms capable of embedding clinical reasoning, teamwork behaviors, and non-technical skills within a cohesive learning experience [32]. The adaptability of escape rooms to both the physical and virtual formats has enlarged their applicability within a range of institutional contexts, particularly as healthcare education increasingly incorporates hybrid or technology-enhanced learning models [33].

The pedagogical potential of escape rooms extends to interprofessional and team-based training. Integrated reviews have emphasized that escape room activities can facilitate shared mental models, role clarity, and coordinated task execution, elements central to safe infection control practices and rapid patient assessment [34]. Their utility has been observed even outside traditional curricular structures, including recruitment events and orientation exercises designed to familiarize participants with organizational procedures and expectations [35]. This flexibility has positioned escape rooms as multipurpose educational tools capable of addressing cognitive, behavioral, and cultural dimensions of safety training.

The emerging work in gamification underlines the fact that such interventions may influence not only knowledge but also learners' clinical reasoning strategies and error detection capabilities [36]. In particular, escape room designs incorporating formative assessment elements, structured debriefing, or scaffolded feedback loops seem to offer special promise in reflective practice and reinforce decision pathways given uncertainty [37]. Similarly, digital and online adaptations of escape rooms have extended opportunities for remote learners in developing critical thinking, problem-solving, and collaborative competencies, for which parallel benefits may also be offered from their physical counterparts [38].

Qualitative examination of learners' experiences has revealed emotional, motivational, and identity-forming influences of participating in escape rooms beyond cognitive benefits. Students consistently report increased engagement, building confidence, and a better understanding of the interplay between technical and non-technical skills in the performance of clinical tasks [39]. In settings focused on the preparation for clinical transition, escape room-based assessment has also served to simulate the rigors of workplace performance, narrowing the gap between classroom learning and practice expectations [40]. Their adoption in geriatric care, toxicology training, and other specialized areas further serves to illustrate the versatility of the format and its capacity to accommodate a range of different curricular objectives [41-44]. 

The escape room contributes to teamwork development, which is particularly relevant as education in healthcare increasingly focuses on interprofessional competencies. Research that embeds collaborative diagnostic reasoning or shared procedural tasks within the settings has demonstrated that gamified contexts foster communication, distributed cognition, and mutual monitoring of performance, essential components of safe care [42]. Additionally, the intentional design of puzzles and challenges can include authentic means for measuring learners' integration of non-technical competencies, to complement traditional metrics of performance [43,45].

Limitations

This review was limited by the predominance of non-randomized and uncontrolled study designs that heighten susceptibility to confounding and hinder causal inference. Substantial heterogeneity in intervention formats, learner populations, and outcome measures reduced cross-study comparability and contributed to imprecision in pooled estimates. Behavioral and process outcomes were drawn from quality improvement contexts in which concurrent institutional initiatives may have influenced results.

Despite these limitations, the present review has several important strengths. This study employed a comprehensive, systematic search strategy across multiple major databases, thereby increasing the likelihood of capturing all relevant studies evaluating escape room-based educational interventions related to infection prevention and patient safety. The review followed established methodological frameworks, including the PECOS structure and PRISMA reporting guidelines, to ensure transparency and methodological rigor. In addition, risk of bias was assessed using validated design-specific tools, and the certainty of evidence was evaluated using the GRADE approach. When sufficient quantitative data were available, a random-effects meta-analysis was conducted to synthesize outcomes across heterogeneous study designs. Together, these methodological approaches strengthen the reliability of the findings and provide a structured synthesis of the current evidence regarding the effectiveness of escape room-based learning in healthcare education.

Clinical recommendations and future implications

Future studies evaluating escape room-based training should use controlled or randomized designs to strengthen causal inferences. Standardization of outcome measures, particularly for knowledge retention, behavioral adherence, and teamwork performance, would contribute to comparability and allow higher-quality meta-analytic synthesis. Objective behavioral or performance-based metrics, complementary to self-report scales, should be incorporated into studies with a view to enhancing measurement precision. Longer-term follow-up is warranted to establish whether gained knowledge translates into sustained behavioral reliability. Implementation research into optimal design features, such as puzzle structure, debriefing strategies, and facilitator roles, may contribute to the refinement of educational applications in infection prevention and patient safety settings.

## Conclusions

This review suggests that escape room-based education results in measurable gains in immediate knowledge in infection prevention and patient safety competencies; longer-term retention of knowledge and behavioral impact are less certain due to methodological limitations and inconsistent findings. The intervention seems educationally worthwhile, but the evidence base is insufficient to make confident claims about generalizability regarding impacts on broader safety behaviors or clinical processes. Further high-quality, well-designed studies are needed to clarify the effects and practical implications of this educational strategy.
